# News and Events of the Week

**Published:** 1909-12-04

**Authors:** 


### NEWS AND EVENTS OF THE WEEK.

THE Medical School of King's College Hospital (separated by the King's College, London (Transfer) Act) has been admitted as an independent body to the status of a school of the University in the Faculty of Medicine.

SIR DONALD MACALISTER, as president, occupied the chair at the 90th session of the General Medical Council at its Oxford Street offices. In the course of his remarks he referred to the proposals for legislation on the subject of anæsthetics, and said they were not likely to make progress this session.

MAJOR-GENERAL LORD CHEYLESMORE, chairman of the Brompton Hospital, opened an exhibition organised by the National Association for the Prevention of Consumption in the Chelsea Town Hall on Tuesday. The exhibition will remain open till to-day, and evening lectures will be given on tuberculosis.

AT the meeting of the General Medical Council, held under the presidency of Sir Donald MacAlister on November 26, the following resolution was carried: "That the regulations of the licensing bodies should be so framed as to secure that the study of the final subjects in the medical curriculum shall be pursued throughout a period not less than two years (24 months) after the examination in anatomy and physiology has been passed."

A SCHEME for the establishment of a Middlesex Sanatorium has been discussed at Hillingdon Court, Uxbridge. It was stated, in the course of the proceedings, that last year there were 1267 deaths from tuberculosis in Middlesex, 899 being from phthisis, and that many district medical officers had expressed themselves in favour of the formation of a county sanatorium. But some of the local authorities affirmed that the necessary money could not be obtained in Middlesex. Colonel Gerard Clark suggested the formation of a twenty thousand crowns fund to provide the first instalment of thirty beds, with an administrative block. Ultimately it is hoped to have a hundred beds in the institution.

A SPECIAL general meeting of the members of the London and Counties Medical Protection Society, Limited, was held on Wednesday, November 24, 1909, at four o'clock in the afternoon, at 31 Craven Street, Strand, London, W.C., for the purposes of considering a resolution "to approve the action of the Council in arranging for an insurance of the members of the Society against any damages or costs up to several thousand pounds which may be awarded at any time against any member in any proceedings which the Society may undertake on behalf of its members pursuant to Article 16 of the Articles of Association of the Society, and for that purpose to raise the subscription of the members of the Society to a sum not exceeding £1 per annum." A large number of the members of the above Society have already effected individual insurance against damages and costs in defensive actions only, and it is now thought desirable to insure the whole of the members of the Society collectively. The insurance can be effected much more satisfactorily when the Society is insured *en bloc*, and if the above scheme is carried through a member of the Society will be indemnified against any loss whether he succeeds or fails in any action, provided only that his case is undertaken by the Council of the Society.

THE annual general meeting of constituents of the Hospital Sunday Fund will be held on Friday, December 17 next. Hospital Saturday falls on June 12 next year.

THE next session of graduate lectures and demonstrations organised by the Berlin Dozenten Vereinigung commences on February 28, and ends on March 26 next. Further particulars may be obtained on application to Messrs. Melzer, Ziegelstrasse 10/11 (Langenbeck House), Berlin.

